# Staff Resources in Public and Private Hospitals and Their Implication for Medical Practice: A French Study of Caesareans

**DOI:** 10.3390/healthcare12101007

**Published:** 2024-05-14

**Authors:** Saad Zbiri, Patrick Rozenberg, Carine Milcent

**Affiliations:** 1 Research Unit 7285 RISCQ, UVSQ, Paris-Saclay University, 78180 Montigny-le-Bretonneux, France; 2 Department of Obstetrics and Gynecology, American Hospital of Paris, 92200 Neuilly-sur-Seine, France; 3 UVSQ, Inserm, Team U1018, Clinical Epidemiology, CESP, Paris Saclay University, 78180 Montigny-le-Bretonneux, France; 4 Paris-Jourdan Sciences Economiques, French National Centre for Scientific Research (CNRS), 75014 Paris, France; 5 Paris School of Economics (PSE), 75014 Paris, France

**Keywords:** staff, workforce, human resource, hospital, public, private, caesarean, France

## Abstract

This study aimed to investigate the effect of hospital staffing resources on medical practice in public versus private hospitals. We used exhaustive delivery data from a French district of 11 hospitals over an 11-year period, from 2008 to 2018, including 168,120 observations. We performed multilevel logistic regression models with hospital fixed or random effects, while controlling for factors known to influence obstetric practice. We found that hospital staff ratios of obstetricians and that of midwives affected caesarean rates, but with different effects depending on the hospital sector. In public hospitals, the higher the ratio of obstetricians and that of midwives, the lower the probability of planned caesareans. In private hospitals, the higher the ratio of obstetricians, the greater the probability of planned caesareans. Indeed, in public hospitals, obstetricians and midwives, both salaried employees, do not have financial or organizational incentives to perform more caesareans. In private hospitals, obstetricians, who are independent doctors, may have such incentives. Our results underline the importance of having an adequate supply of health professionals in healthcare facilities to ensure appropriate care, with specific regard to the different characteristics of the public and private sectors.

## 1. Introduction

The provision of health services is dependent on a complex array of medical, economic, social, and political characteristics [1]. The country requirements for health services, including in terms of human resources, may vary according to population age, sex, density, and mortality, education and wealth, geographic environment, patterns of use of health services, and type of access to these services [2].

Most countries, whether low- or high-income, are facing a growing demand for health services and increasing healthcare costs. Their funding for health services is limited, and they are constantly looking to make efficient use of health resources [1]. In addition, current resources in health systems should first be reallocated fairly to allow an equitable redistribution of existing resources within healthcare systems [3]. The health workforce is an absolutely key resource, consuming a large proportion of the healthcare budget, and with characteristics such as skills, abilities, or commitment that may impact the effectiveness and efficiency of healthcare delivery [4].

Health facilities, both public and private, are expected to improve the quantity, quality, and accessibility of the healthcare services they provide, while having to operate within limited financial resources [5]. Thus, one important issue to examine is the allocation of staff resources in health centers, as well as how they can be used more effectively to enhance patient outcomes [6]. Indeed, the impact of the structure of the hospital staffing on medical practices should be studied, in order to establish, if necessary, appropriate staffing standards for hospitals.

Caesarean delivery is one of the most widely performed surgical procedures in the world. Rates of caesareans have risen continuously over the last few decades, with some countries exceeding a 50% rate, while the World Health Organization recommends for all countries a rate of between 10% and 15% [7]. This overuse of caesareans is a major public health issue. Indeed, compared with normal deliveries, caesareans are associated with higher financial costs [8], as well as increased risks of morbidity and mortality for the mother, while there are no health benefits for the newborn [9].

The clinical characteristics of women do not explain all indications for caesareans. Non-clinical factors may contribute to the need for a caesarean. These include the patient’s socioeconomic characteristics [10], the woman’s choice [11], or the doctor’s incentives [12]. Hospital characteristics also have an effect [13]. Previous studies have shown that hospital ownership [14], equipment levels [15], teaching status [16], and hospital size all have an impact on the use of caesareans [17]. 

In addition, several studies have reported an effect of hospital staff on caesarean rates. First, some characteristics of medical staff have an impact on the use of caesareans. A systematic review and meta-analysis showed that female doctors are less likely to perform caesareans, and less likely to prefer them [18]. Another study found that women delivered by obstetricians with a low volume of deliveries run a significantly higher risk of caesarean [19]. The type of health provider is also a key factor. Care provided by obstetricians, compared with that provided by midwives, is associated with an increased risk of caesarean, particularly for low-risk women [20,21]. Additionally, hospitals with integrated midwifery services are associated with lower caesarean rates [22,23]. The organization of hospital staff practice also plays a significant role. A practice model with a laborist doctor, who is continuously present within the obstetric department to supervise the management of labor and deliveries, is associated with lower caesarean rates than a private practice model [24,25]. Other studies have observed that women who gave birth in hospitals where obstetricians were available on call had lower rates of intrapartum caesarean than other women [26,27]. Finally, some recent studies have suggested an effect of hospital staffing levels on caesarean rates. These studies analyzed staffing levels for obstetricians, anesthetists, or midwives, as estimated by the staff number reported to the activity of the obstetric unit. However, differences between the public and private sectors were not regularly considered in these analyses [28,29].

The aim of this study was to examine the effect of hospital staffing ratios on caesarean rates. Our study therefore reinforces the existing scientific literature regarding the association between human resources staffing and medical practice. In particular, it provides an opportunity to investigate this relationship in both the public and private sectors. Based on the literature review, we developed two hypotheses to be tested in the present study. First, we hypothesized that hospital staffing ratios may affect caesarean rates. Second, we hypothesized that this impact may differ between public and private hospitals. Using exhaustive delivery data from a French district of 11 hospitals over several years, we performed multilevel logistic regression models while controlling for the common characteristics of women and hospitals to analyze the effect of hospital staff ratios on caesarean use in both public and private maternity hospitals.

In the next section, we describe the data and the statistical method used for the study. The third section reports the results and robustness checks. In the fourth section, we interpret our results and present the study advantages and limitations. In the final section, we provide a general conclusion.

## 2. Materials and Methods

### 2.1. Data

We carried out a French retrospective population study. We used data on deliveries from 2008 through 2018 from two databases. One database included all deliveries in the Yvelines district. The second database provided information about all French hospitals. As caesarean determinant factors are not generally included in a single dataset, we used these two databases in order to consider the individual and hospital factors that may have an impact on the use of caesareans. The first database was compiled from the first health certificate of infants born in the Yvelines district. In France, this certificate is completed for each newborn before discharge from the hospital. This first database provided demographic information on the woman; information on the pregnancy, including medical support and hospital stays; information on the delivery, including hospital of delivery, date of delivery, mode of delivery, and delivery procedures; and comprehensive information on maternal, fetal, and neonatal health, including all diagnoses and comorbidities. All births in the district during the full calendar years from 2008 to 2018 were considered. Stillbirths, terminations of pregnancy, and births that took place outside hospitals were deleted. The data were double-checked to address incorrect and missing information. The final missing data rates were <3% for all variables. The study sample consisted of 168,120 deliveries. The second database was the French annual statistics for hospitals. This survey, performed by the Ministry of Health, provided information on all hospitals, including their location, sector, level of equipment, volume of activity, and the composition of hospital staff. Information on hospitals with obstetric care in the Yvelines district was located and merged with the individual data from the first database.

Our data were exhaustive for the Yvelines district during the whole study period (2008–2018). Based on its population, Yvelines is the eighth largest district in France. The study sample was equivalent to around 22% of the annual number of deliveries in France (750,000 on average) [30]. In addition, our data concerned 11 hospitals with different characteristics, covering all types of hospitals in France. We also used a decade-long dataset in order to consider regular changes in hospital characteristics from one year to the next, especially those relating to staff ratios, which was a major advantage of our study. We can thus consider that our data may provide a close approximation of the situation in France.

### 2.2. Variables

The variable of interest was the caesarean binary variable. Our exclusive French data allowed us to consider the common factors that influence caesareans, including the woman’s demographics, her clinical risk factors, and a set of hospital characteristics, including hospital sector and staff characteristics. Indeed, we had access to demographic variables, including age and parity; clinical risk variables, including previous caesarean, diabetes, hypertension, eclampsia or preeclampsia (including HELLP syndrome), intrauterine growth restriction, placental disorder (including placenta previa, placenta accreta, and abruptio placenta), other obstetric pathology (such as obesity, infection, premature rupture of membranes, amniotic fluid abnormality, or congenital anomaly), multiple pregnancy, preterm delivery (gestational age < 37 weeks), post-term delivery (gestational age > 41 weeks), abnormal fetal presentation (breech or transverse lie), induced labor, low birth weight (<2500 g), and high birth weight (>4000 g); variables for the hospital type, including hospital sector, equipment level, and university status; variables for the hospital organization, including the availability of obstetricians 24 h a day, on the day of delivery, and the size of the unit in relation to the annual volume of deliveries; and variables for the hospital staff, including the ratios of obstetricians, of anesthetists, and of midwives. 

Hospital staffing ratios were estimated as the number of health workers in proportion to hospital output. All hospitals declared their staff in full-time equivalent terms, using available information on hours worked. These data were comparable for all hospitals. We calculated the average full-time equivalents per year for each staff group using all employees in that group present in the hospital. We checked that all obstetricians, anesthetists, and midwives present in the maternity unit were actually involved in obstetric practice. The presence of trainees, such as residents and interns, was not continuous, and their numbers, not available with accuracy, were not considered. Indeed, they all worked under the supervision of a senior doctor, and the university status variable made it possible to control for the difference between hospitals receiving trainees and other hospitals. Doctors in private hospitals were reported in numbers of employees only, as they were self-employed, and their work time was not known. To capture the effective rates of practice of private doctors who were working part-time, we applied the default assumption that these employees were in the hospital for 50% of their time. However, we also considered two extreme assumptions of 25% and 75% as sensitivity checks to verify the reliability of our results. The weighted values were then added to the full-time equivalent numbers. Various previous studies had already applied this method [28]. To estimate the output of the hospital, we referred to the total number of deliveries per year, which was divided by 100 to produce easier-to-read estimates. However, as robustness analyses, further indicators were also used, including the number of mean occupied beds and the number of hospital stays.

### 2.3. Statistical Analysis

The empirical analysis used panel data to examine the effect of hospital staffing ratios on the use of caesareans. The dependent variable was the caesarean dummy variable. The independent variables were demographic characteristics, clinical risk factors, hospital type, organization, and staff variables, as listed above.

Our models were performed using multilevel logistic regressions and estimated standard errors robust to heteroskedasticity that accounted for within-hospital dependencies between observations. We used logistic regression because our outcome variable (caesarean) was a binary variable with values of 0 or 1. Logistic regression is a statistical model used to study the association between a response (dependent) variable Yi and a set of explanatory (independent) variables Xi [31]. As our panel was composed of women who gave birth in different years and hospitals, we included fixed effects for year of delivery and for hospitals in each model in order to consider heterogeneity over time and between hospitals. We also used random hospital effects to consider invariant hospital characteristic variables in the multilevel logistic models, such as the hospital sector or the university status. A variable was considered significant if the result was significant in both the hospital fixed-effects model, as well as in the hospital random-effects model.

Our models included all available variables known in the literature to significantly influence the use of caesareans, in order to take into account correlations between all variables. Variables for hospital staff were crossed with the hospital sector variable in order to consider differences between hospital sectors. Obstetricians are salaried in the public sector, whereas in the private for-profit sector they are self-employed. In contrast, anesthetists and midwives are generally salaried, regardless of the hospital sector. The organization of care also differs between public and private hospitals. In addition, as the impact of hospital staff may differ according to the type of delivery, we studied all caesareans together, but also planned and unplanned caesareans separately. We also analyzed the entire population, as well as the high-risk population and the low-risk population, which were defined according to the commonly used clinical criteria [32]. The results are presented as coefficients with their standard errors in parentheses. Each result with a two-tailed *p*-value of less than 0.050 was considered significant. We used Stata software (https://www.stata.com) for all analyses [33].

### 2.4. Ethics and Legislation

The dataset was managed by the district council of Yvelines (*conseil départemental des Yvelines*), in partnership with the regional health agency of Ile-de-France (*agence régionale de santé de l’Ile-de-France*), and the local perinatal network of Yvelines (*réseau périnatal maternités en Yvelines et périnatalité active*). The data used have been declared to the French data protection authority (*commission nationale de l’informatique et des libertés*), under number 1295794. All data did not contain sensitive or identifiable information, and were allowed to be used for standard analysis. Ethical approval was therefore not required, as stated by French law. 

## 3. Results

### 3.1. Descriptive Results

This study covered 168,120 deliveries that took place in the Yvelines district from 2008 to 2018. Table 1 presents general statistics on the patient and hospital characteristics of women giving birth in the district. The average patient age was 31 years, and 42% of the women were nulliparous. Induced labor, previous caesarean, and other obstetric pathologies were the most prevalent clinical risk factors for caesarean, which occurred in 22%, 11%, and 7% of all women, respectively.

There were 11 hospitals providing obstetric care in the Yvelines district, including five public hospitals and six private hospitals. Overall, public hospitals accounted for 67% of deliveries, while private hospitals accounted for 33% of deliveries. The number of deliveries remained almost unchanged from 2008 through 2018. Hospitals with no special neonatal care accounted for 18% of deliveries, hospitals with special neonatal care for 39% of deliveries, and hospitals with intensive neonatal care for 43% of deliveries. University hospitals were responsible for 39% of deliveries.

In terms of hospital organization, 26% of deliveries were carried out on non-working days, including weekends and public holidays, and 13% of deliveries took place with no obstetrician present in hospital for 24 h. Small hospitals accounted for 15% of deliveries, medium-sized hospitals for 30% of deliveries, and large hospitals for 55% of deliveries. In terms of hospital staff, the average number of obstetricians per delivery was 0.50, fairly close to that of anesthetists which was 0.56, but less than that of midwives, which was 1.63.

Over the period 2008–2018, the average caesarean rate was 24.5% in the Yvelines district, with almost 23% in public hospitals and 28% in private hospitals. This average rate remained relatively stable throughout the period studied.

### 3.2. Regression Results

Table 2 shows the effect of hospital staffing ratios in public versus private hospitals on caesarean use among the overall population (168,120 observations). Controlling for demographic, clinical, and hospital characteristics in columns 1 and 2, we found no significant effect regardless of the hospital staff category. However, when we focused on planned caesareans, as presented in columns 3 and 4, we observed a significant effect. Obstetrician and midwife staffing ratios impacted the probability of planned caesareans. This effect of obstetricians and midwives was apparent in public hospitals, but not in private hospitals. Indeed, irrespective of individual- and hospital-level characteristics, we observed that the higher the ratio of obstetricians, the lower the planned caesarean rate (coefficient = −0.620, robust standard error = 0.228, *p*-value = 0.007, for the hospital random effects model; and coefficient = −0.659, robust standard error = 0.200, *p*-value < 0.001, for the hospital fixed effects model). Similarly, the higher the ratio of midwives, the lower the planned caesarean rate (coefficient = −0.191, robust standard error = 0.063, *p*-value = 0.002, for the hospital random effects model; and coefficient = −0.197, robust standard error = 0.076, *p*-value = 0.010, for the hospital fixed effects model). Finally, when we looked at unplanned caesareans, as reported in columns 5 and 6, we found no significant effect, whatever the category of hospital staff.

We then studied the effect of hospital staffing ratios on caesarean use across different clinical women groups, in high and low medical risk subgroups. On the one hand, Table 3 reports this effect in the high-risk subgroup (82,850 observations). The high clinical risk population consisted of those women who had at least one diagnosis or comorbidity that increased the probability of caesarean. In columns 3 and 4, all else being equal, the effect of staffing ratios for obstetricians in public hospitals on planned caesareans was still significant (coefficient = −0.848, robust standard error = 0.277, *p*-value = 0.002, for the hospital random effects model; and coefficient = −0.779, robust standard error = 0.324, *p*-value = 0.016, for the hospital fixed effects model).

On the other hand, Table 4 presents the effect of hospital staffing ratios on the use of caesareans in the low-risk subgroup (33,370 observations). As generally defined in the literature, our low clinical risk subsample consisted of nulliparous women aged between 20 and 34 years old, with no diagnosis or comorbidity, delivering at term, without induction, a single child in cephalic presentation, with a normal birth weight [15]. In columns 3 and 4, independently of other factors observed, we found a significant impact of obstetrician ratios on the rate of planned caesareans in private hospitals. Indeed, for low-risk women giving birth in private hospitals, the higher the ratio of obstetricians, the higher the planned caesarean rate (coefficient = 1.486, robust standard error = 0.406, *p*-value < 0.001, for the hospital random effects model; and coefficient = 1.051, robust standard error = 0.390, *p*-value = 0.007, for the hospital fixed effects model).

### 3.3. Additional Results and Sensitivity Checks

Table S1 in the Supplementary Materials presents the effect of individual and hospital factors, used as control variables in our logistic regression models, on the use of caesareans. Age and nulliparity increased the caesarean probability. As expected, well-known obstetric risks affected the mode of delivery. Caesareans were more common in high clinical risk patients than in low clinical risk women. Abnormal presentation, previous caesarean, and placental disorder were the greatest medical risks. Admission to a private hospital, compared with a public hospital, increased the probability of caesareans. Maternity units with neonatal intensive care, versus those with no special neonatal care, had an increased probability of caesareans. Delivery on a non-working day was negatively associated with caesareans. Finally, giving birth in large maternity units, compared with medium-sized maternity units, decreased the probability of caesareans. In summary, different individual and institutional characteristics were significant predictors of caesareans, in accordance with findings from the literature.

To assess the robustness of our empirical findings, we performed sensitivity analyses. First, to address the heterogeneity of information on hospital staff between public and private hospitals, we considered an average situation in which self-employed doctors working part-time devoted 50% of their time to their hospital work. To ensure that this weighting we considered did not affect the results, we suggested two extreme cases. A so-called “minimalist” level of work, in which these doctors only devoted 25% of their time, and a so-called “maximalist” level of work, obtained by applying a coefficient of 0.75 to the total. We found similar results regardless of the weighting used to evaluate the time spent by private practice doctors in hospitals (Tables S2–S7 in the Supplementary Materials).

Second, hospital staffing ratios were estimated based on the number of obstetricians, anesthetists, and midwives proportional to the production of the maternity unit. To estimate the production of each unit, we used the number of deliveries in total per year. As a robustness check, we also used two other indicators: the number of mean occupied patient beds per year, and the number of hospital stays in total per year. The mean occupied bed figure was calculated from the length of stays and the number of beds in each unit. The number of deliveries initially used could focus on the delivery production while being specific; however, the number of mean occupied beds and the number of hospital stays could consider the whole production of the maternity unit while being less specific. Results remained the same, confirming that our findings were robust and conservative. Full tables of sensitivity analyses are available upon request.

## 4. Discussion

### 4.1. Main Findings

Using exhaustive and large delivery data from a French district of 11 hospitals over an 11-year period, our results showed that the ratio of obstetricians and that of midwives affected caesarean rates. This hospital staff effect was observed for planned caesareans and differed between public and private hospitals.

### 4.2. Interpretation

In public hospitals, the higher the ratio of obstetricians and that of midwives, the lower the probability of planned caesareans. This result was observed in the context of public sector hospitals, where the hospital staff are salaried employees of the establishment. Staff salaries do not depend directly on the type or volume of the hospital activity. There is therefore no financial incentive for doctors and midwives to perform more caesareans in these hospitals [12]. Several hypotheses may explain this result. Having more obstetricians and midwives per woman could increase the availability of hospital staff. This could lead to better medical follow-up for women, as well as greater information for them, both during pregnancy and childbirth. Several studies have shown that increased medical support for women reduces maternal and fetal complications, and therefore leads to a lower risk of caesarean [34]. Improved education and information for women during pregnancy could also reduce caesarean rates [35]. In addition, a high proportion of medical staff might improve collaboration among staff members at the maternity unit, with greater involvement in setting up clinical protocols for good practice and participation in unit staff meetings. Recent studies have shown that hospitals with a policy of discussion and validation of caesarean indications have reduced caesarean rates [36]. Finally, a greater availability of hospital staff could reduce the risk aversion associated with medical practice, by providing closer monitoring to women at risk of caesarean, and thus avoiding a caesarean when possible. Indeed, defensive medicine is an important factor in increasing the probability of having a caesarean, particularly for doctors, who handle high-risk pregnancies and are generally much more liable to expect potential complications [37].

In private hospitals, the higher the ratio of obstetricians, the higher the probability of planned caesareans. This result applies to private hospitals where doctors have a private practice and are paid on a fee-for-service basis. Private obstetricians have a financial incentive to perform more caesareans, since when a caesarean is performed, the total amount, including extra fees, charged to the woman is greater [12]. Having a low number of women per doctor could reduce the income of doctors and lead to more caesareans. This result has already been reported in several studies, showing that doctors adapt their caesarean practice to variations in the number of women they support, in a context where caesareans are much more profitable than normal deliveries [38]. In addition to financial incentives, other types of incentives, particularly organizational incentives, may explain this result. Indeed, the private practice could encourage the use of more caesareans. Private obstetricians operate between their own office and the private hospital, and the use of planned caesareans could help to organize their medical activity [39]. This result regarding private obstetricians was observed in women at low risk and who therefore showed no formal indication for a caesarean. This confirms the impact of financial and organizational characteristics, rather than clinical factors. The result regarding midwives is also important to discuss. In the private hospital sector, midwives are salaried employees, as they are in the public sector. They therefore have no direct financial or organizational incentives in private hospitals. Unlike for doctors, our results showed a negative correlation between the ratio of midwives and planned caesareans. Although the coefficients were not significant at 5%, they were significant at 10% for the total population of women, both in the main and robustness analyses. This clearly shows that the effect of the staff ratio depends much more on incentives and may even differ within the same sector. This low significance of the effect of midwives in the private sector as compared to the public sector can be explained by a difference in the organization of care where women in private maternity hospitals are mainly allocated to a single obstetrician, whereas midwives in public hospitals actively follow women during their pregnancy.

### 4.3. Implication and Future Perspectives

Human resources are a key component of healthcare systems worldwide. Their regulation in the healthcare market and their management in hospitals are major areas of concern. Meanwhile, there are ongoing challenges in terms of adequacy and quality of care, particularly with regard to the difficulty of promoting quality in all healthcare organizations. Our results highlight the significant impact of staff resources on medical practice, with different effects depending on the hospital sector. In the public sector, where there is no financial or organizational incentive to perform more caesareans, we found a favorable effect of obstetrician and midwife proportions on the relevance of care. However, in the private sector, where there may be financial and organizational incentives, we observed an adverse effect of obstetrician proportion on the adequacy of care.

Our study may have a number of implications for policy makers and hospital managers. For policy makers, our results should encourage reflection on minimum staffing levels needed to ensure the quality and safety of care. Our results should also provide further support for the consideration of financial and organizational incentives that may lead to inappropriate medical practices. The DRG-based payment used in most high-income countries encourages hospital performance, but does not always allow the quality of care to be sufficiently considered. Financing for quality of care could encourage hospitals to ensure greater relevance and optimal quality of care for the benefit of patients and society [40]. For hospital managers, our results are of major importance for hospitals with high caesarean rates as they seek effective solutions to reduce unnecessary caesarean practices. Indeed, analyzing the staffing resources of maternity units, identifying the different incentives that could have an impact on hospital staff, and considering the resulting implications for medical practice, may all help reduce caesarean rates.

Our results could be enhanced by future research on medical practices outside obstetric care, as well as on health services outside hospitals, such as community or private practice care. They may also be supplemented by further analyses conducted in other aspects of healthcare and in other country settings. Other types of studies, in particular experimental and quasi-experimental studies, are desirable to confirm our findings. Notably, examining the impact of changes in resource allocation on the adequacy and quality of care should be given greater priority.

### 4.4. Strengths and Limitations

Our study had several strengths. First, we used exhaustive data from a French district with 11 hospitals providing obstetric care. The number of observations was high, with over 160,000 deliveries, which helped us obtain statistically reliable results. Our data also covered a recent and large period of 11 years, producing results that are up-to-date and consistent over time. Second, our dataset provided extensive information, allowing us to control for a wide range of determinant factors likely to have an impact on the use of caesareans, including demographic variables, clinical risk variables, as well as hospital type, organization, and staff variables. We were also able to carry out an in-depth analysis, considering the different types of caesarean, both planned and unplanned caesareans, and the different levels of clinical risk of the women in both high-risk and low-risk subgroups. Finally, the quality of the data was also a major advantage. The individual data from the first health certificates were filled in by doctors and midwives, almost prospectively from birth. The contents of these certificates were double-checked to correct inaccurate and missing information. In addition, hospital information from the French annual statistics for hospitals was checked and supplemented by data from the local perinatal network. The overall information extracted was therefore of high quality.

Our study may have had limitations. On the one hand, our data covered the Yvelines district, and not the French national situation. However, the number of deliveries in our study accounted for around 22% of the annual births in France [30]. Moreover, the characteristics of the hospitals in Yvelines, with 11 hospitals both public and private, and with different characteristics in terms of equipment, teaching and size, were very similar to those of other hospitals in all of France. In particular, the distribution of deliveries by sector in the Yvelines was similar to the national distribution, and the statistics on hospital staff ratios in the Yvelines were very similar to the French national situation, as reported in the annual statistics for French hospitals. Our results may then be applied to other geographical areas of France, outside the district of Yvelines. On the other hand, although we took into account several factors affecting the use of caesareans, we were unable to be exhaustive about all of the determinant factors. However, we did have access to comprehensive information on the medical severity of the mother and her fetus, and to a large set of information on hospitals. Indeed, we included all available determinant variables in the final models, and also conducted multilevel models to control for unobservable hospital-related factors. Hospital fixed-effects models allowed us to consider potential correlations between hospital characteristics not explicitly accounted for in the models and any independent variables, and hospital random-effects models enabled us to take account of all variables, including time-invariant hospital variables, while controlling for unobservable hospital effects in the random part of the model. Lastly, our analyses assumed that private doctors working part-time were operating at 50% of a full-time equivalent. To check whether this weighting of part-time staff affected the results, we also considered two more extreme cases, at 25% and 75%, and obtained very similar results.

## 5. Conclusions

Our study revealed a significant impact of hospital staff ratios on caesarean rates, including for obstetricians and midwives. However, this effect of hospital staffing differed between public and private hospitals. The staff allocation in hospitals therefore seems to influence the appropriateness of care. Policy makers as well as hospital managers need to take account of hospital sector-specific staffing resources and their effect on medical practice.

## Figures and Tables

**Table 1:** Descriptive statistics. All population.

Sample: All Population		
	*n*	Percent (Mean)
Outcome variable		
Caesarean	41,107	24.45
Demographic variables		
Age (years)	168,120	30.96 (5.15)
Nulliparous	70,019	41.65
Clinical risk variables		
Previous caesarean	18,654	11.10
Diabetes	8829	5.25
Hypertension	1961	1.17
Eclampsia/Preeclampsia	1503	0.89
Fetal growth restriction	2390	1.42
Placental disorder	484	0.29
Other pathology	11,852	7.05
Multiple pregnancy	2720	1.62
Preterm delivery	9084	5.40
Post-term delivery	253	0.15
Abnormal presentation	6952	4.14
Induced labor	37,053	22.04
Low birth weight	9693	5.77
High birth weight	11,598	6.90
Hospital type variables		
Private	55,436	32.97
Level of equipment		
No neonatology unit	30,365	18.06
Neonatology unit	65,016	38.67
Neonatal intensive unit	72,739	43.27
University	64,763	38.52
Hospital organization variables		
On-call obstetrician	21,673	12.89
Non-working day	44,409	26.42
Size		
Small	25,281	15.04
Medium	50,062	29.78
Large	92,777	55.18
Hospital staff variables		
Obstetricians	168,120	0.50 (0.14)
Anesthetists	168,120	0.56 (0.23)
Midwives	168,120	1.63 (0.44)

Data source: Yvelines district (France), 2008–2018. Notes: Means are given with their standard deviation in parentheses for continuous variables including age, obstetricians, anesthetists, and midwives.

**Table 2:** Effect of hospital staffing ratios on caesareans. Multilevel logistic models (coefficient estimates). All population.

Sample: All Population						
Dependent Variable	Caesarean		Planned Caesarean		Unplanned Caesarean	
	(1)	(2)	(3)	(4)	(5)	(6)
Crossed hospital staff and hospital sector variables						
Obstetricians × Public	−0.123	−0.108	−0.659 ***	−0.620 **	0.082	0.087
	(0.283)	(0.280)	(0.200)	(0.228)	(0.371)	(0.377)
Obstetricians × Private	0.164	0.156	0.113	−0.058	0.040	0.158
	(0.180)	(0.185)	(0.262)	(0.284)	(0.200)	(0.143)
Anesthetists × Public	0.345	0.360	0.069	0.094	0.361	0.375
	(0.248)	(0.251)	(0.192)	(0.195)	(0.263)	(0.266)
Anesthetists × Private	−0.007	−0.007	0.009	0.109	−0.037	−0.104
	(0.138)	(0.147)	(0.159)	(0.156)	(0.162)	(0.164)
Midwives × Public	−0.118	−0.123	−0.191 **	−0.197 **	−0.021	−0.027
	(0.170)	(0.165)	(0.063)	(0.076)	(0.210)	(0.203)
Midwives × Private	0.081	0.062	−0.182	−0.173	0.254	0.221
	(0.112)	(0.114)	(0.097)	(0.104)	(0.170)	(0.161)
Control variables						
Demographics	Yes	Yes	Yes	Yes	Yes	Yes
Clinical risks	Yes	Yes	Yes	Yes	Yes	Yes
Hospital type	Yes	Yes	Yes	Yes	Yes	Yes
Hospital organization	Yes	Yes	Yes	Yes	Yes	Yes
Multilevel effects						
Year effects	Fixed	Fixed	Fixed	Fixed	Fixed	Fixed
Hospital effects	Random	Fixed	Random	Fixed	Random	Fixed
Observations	168,120	168,120	168,090	168,090	150,226	150,226

Data source: Yvelines district (France), 2008–2018. Notes: ** *p*-value < 0.01; *** *p*-value < 0.001. Robust standard errors clustered at the hospital level in parentheses. Control variables included for demographics: age and parity; for clinical risks: previous caesarean, diabetes, hypertension, eclampsia or preeclampsia, fetal growth restriction, placental disorder, other obstetric pathology, plurality, term at delivery, fetal presentation, induced labor, and birth weight; for hospital type: sector, equipment level, university status; and for hospital organization: obstetrician availability, day of delivery, and size.

**Table 3:** Effect of hospital staffing ratios on caesareans. Multilevel logistic models (coefficient estimates). High-risk population.

Sample: High-Risk Population						
Dependent Variable	Caesarean		Planned Caesarean		Unplanned Caesarean	
	(1)	(2)	(3)	(4)	(5)	(6)
Crossed hospital staff and hospital sector variables						
Obstetricians × Public	−0.227	−0.187	−0.848 **	−0.779 *	0.069	0.082
	(0.243)	(0.252)	(0.277)	(0.324)	(0.302)	(0.322)
Obstetricians × Private	0.209	0.167	−0.194	−0.488	0.179	0.358 **
	(0.145)	(0.174)	(0.293)	(0.306)	(0.165)	(0.137)
Anesthetists × Public	0.238	0.281	0.033	0.089	0.235	0.272
	(0.232)	(0.231)	(0.204)	(0.208)	(0.257)	(0.257)
Anesthetists × Private	0.059	0.071	0.119	0.294 *	0.048	−0.082
	(0.101)	(0.113)	(0.189)	(0.144)	(0.120)	(0.120)
Midwives × Public	−0.007	−0.020	−0.131 *	−0.143	0.092	0.080
	(0.152)	(0.141)	(0.067)	(0.075)	(0.198)	(0.188)
Midwives × Private	0.113	0.080	−0.098	−0.089	0.230	0.175
	(0.128)	(0.130)	(0.117)	(0.117)	(0.196)	(0.180)
Control variables						
Demographics	Yes	Yes	Yes	Yes	Yes	Yes
Clinical risks	Yes	Yes	Yes	Yes	Yes	Yes
Hospital type	Yes	Yes	Yes	Yes	Yes	Yes
Hospital organization	Yes	Yes	Yes	Yes	Yes	Yes
Multilevel effects						
Year effects	Fixed	Fixed	Fixed	Fixed	Fixed	Fixed
Hospital effects	Random	Fixed	Random	Fixed	Random	Fixed
Observations	82,850	82,850	82,828	82,828	68,517	68,517

Data source: Yvelines district (France), 2008–2018. Notes: * *p*-value < 0.05; ** *p*-value < 0.01. Robust standard errors clustered at the hospital level in parentheses. Control variables included for demographics: age and parity; for clinical risks: previous caesarean, diabetes, hypertension, eclampsia or preeclampsia, fetal growth restriction, placental disorder, other obstetric pathology, plurality, term at delivery, fetal presentation, induced labor, and birth weight; for hospital type: sector, equipment level, university status; and for hospital organization: obstetrician availability, day of delivery, and size.

**Table 4:** Effect of hospital staffing ratios on caesareans. Multilevel logistic models (coefficient estimates). Low-risk population.

Sample: Low-Risk Population						
Dependent Variable	Caesarean		Planned Caesarean		Unplanned Caesarean	
	(1)	(2)	(3)	(4)	(5)	(6)
Crossed hospital staff and hospital sector variables						
Obstetricians × Public	−0.114	−0.160	0.155	−0.335	−0.118	−0.185
	(0.443)	(0.437)	(1.001)	(1.303)	(0.513)	(0.534)
Obstetricians × Private	0.471	0.580	1.486 ***	1.051 **	−0.110	0.331
	(0.422)	(0.393)	(0.406)	(0.390)	(0.547)	(0.485)
Anesthetists × Public	0.479*	0.423	−0.428	−0.985 **	0.589	0.569
	(0.272)	(0.259)	(0.263)	(0.359)	(0.309)	(0.294)
Anesthetists × Private	−0.207	−0.287	−0.817 *	−0.597	−0.094	−0.341
	(0.231)	(0.238)	(0.386)	(0.374)	(0.198)	(0.226)
Midwives × Public	−0.119	−0.124	0.124	0.404	−0.086	−0.164
	(0.261)	(0.240)	(0.341)	(0.363)	(0.295)	(0.251)
Midwives × Private	0.259	0.167	−0.172	−0.222	0.496	0.329
	(0.233)	(0.231)	(0.206)	(0.242)	(0.312)	(0.279)
Control variables						
Demographics	Yes	Yes	Yes	Yes	Yes	Yes
Clinical risks	Yes	Yes	Yes	Yes	Yes	Yes
Hospital type	Yes	Yes	Yes	Yes	Yes	Yes
Hospital organization	Yes	Yes	Yes	Yes	Yes	Yes
Multilevel effects						
Year effects	Fixed	Fixed	Fixed	Fixed	Fixed	Fixed
Hospital effects	Random	Fixed	Random	Fixed	Random	Fixed
Observations	33,370	33,370	33,369	33,369	32,271	32,271

Data source: Yvelines district (France), 2008–2018. Notes: * *p*-value < 0.05; ** *p*-value < 0.01; *** *p*-value < 0.001. Robust standard errors clustered at the hospital level in parentheses. Control variables included for demographics: age and parity; for clinical risks: previous caesarean, diabetes, hypertension, eclampsia or preeclampsia, fetal growth restriction, placental disorder, other obstetric pathology, plurality, term at delivery, fetal presentation, induced labor, and birth weight; for hospital type: sector, equipment level, university status; and for hospital organization: obstetrician availability, day of delivery, and size.

## Data Availability

Data are available on request for reasons of confidentiality and administrative authorizations. The authors had access to the data for scientific research after receiving the agreement of the Yvelines district council (*conseil départemental des Yvelines*), which is responsible for the data, via the local perinatal network (*MYPA*). The data used have been declared to the French data protection authority (*commission nationale de l’informatique et des libertés*), under number 1295794. Interested researchers can access these data after receiving all the necessary authorizations by contacting the local perinatal network (*MYPA*) at contact@mypa.fr.

## Supplementary Materials

The following supporting information can be downloaded at: https://www.mdpi.com/article/10.3390/healthcare12101007/s1. Table S1. Effect of individual and hospital factors on caesareans. Multilevel logit models (coefficient estimates). All population. Table S2. Effect of hospital staffing ratios on caesareans. Multilevel logit models (coefficient estimates). All population. Assumption of 25% for part-time private doctors. Table S3. Effect of hospital staffing ratios on caesareans. Multilevel logit models (coefficient estimates). All population. Assumption of 75% for part-time private doctors. Table S4. Effect of hospital staffing ratios on caesareans. Multilevel logit models (coefficient estimates). High-risk population. Assumption of 25% for part-time private doctors. Table S5. Effect of hospital staffing ratios on caesareans. Multilevel logit models (coefficient estimates). High-risk population. Assumption of 75% for part-time private doctors. Table S6. Effect of hospital staffing ratios on caesareans. Multilevel logit models (coefficient estimates). Low-risk population. Assumption of 25% for part-time private doctors. Table S7. Effect of hospital staffing ratios on caesareans. Multilevel logit models (coefficient estimates). Low-risk population. Assumption of 75% for part-time private doctors.
