# Supplementary material for: Staff Resources in Public and Private Hospitals and Their Implication for Medical Practice: A French Study of Caesareans
Source: Healthcare (Basel). 2024 May 14;12(10):1007. doi: 10.3390/healthcare12101007 (PMC11120882; doi:10.3390/healthcare12101007)
Supplement: Supplementary file 1 [file healthcare-12-01007-s001.zip › healthcare-2951222-supplementary.pdf]

Supplementary Materials

Table S1. Effect of individual and hospital factors on caesareans. Multilevel logit models (coefficient estimates). All population.

Sample: All population		
**Dependent variable:**	**Caesarean**	**Caesarean**
	(1)	(2)
**Demographic variables**		
Age (years)	0.044***	0.044***
	(0.002)	(0.002)
Nulliparous	1.185***	1.185***
	(0.072)	(0.072)
**Clinical risk variables**		
Previous caesarean	3.050***	3.050***
	(0.108)	(0.108)
Diabetes	0.299***	0.299***
	(0.038)	(0.038)
Hypertension	0.510***	0.509***
	(0.067)	(0.067)
Eclampsia/Preeclampsia	1.215***	1.215***
	(0.089)	(0.089)
Fetal growth restriction	0.575***	0.576***
	(0.148)	(0.148)
Placental disorder	2.339***	2.340***
	(0.230)	(0.230)
Other pathology	0.030*	0.030*
	(0.013)	(0.013)
Multiple pregnancy	0.534***	0.534***
	(0.124)	(0.124)
Preterm delivery	0.469***	0.469***
	(0.098)	(0.098)
Post-term delivery	0.778***	0.778***
	(0.089)	(0.089)
Abnormal presentation	3.510***	3.511***
	(0.108)	(0.108)
Induced labor	0.182*	0.182*
	(0.084)	(0.084)
Low birth weight	0.576***	0.575***
	(0.048)	(0.048)
High birth weight	0.644***	0.644***
	(0.067)	(0.067
**Hospital type variables**		

Private	0.418*	
	(0.165)	
Neonatology unit	0.078	0.110
	(0.070)	(0.067)
Neonatal intensive unit	0.151*	0.169*
	(0.076)	(0.081)
University	0.107	
	(0.169)	
**Hospital organization variables**		
On-call obstetrician	0.041	0.032
	(0.063)	(0.065)
Non-working day	-0.506***	-0.506***
	(0.056)	(0.056)
Small size	-0.214	-0.226
	(0.116)	(0.118)
Large size	-0.101*	-0.109*
	(0.045)	(0.043)
**Hospital staff variables**		
Obstetricians	-0.032	-0.040
	(0.111)	(0.115)
Anesthetists	0.175	0.180
	(0.122)	(0.126)
Midwives	-0.032	-0.040
	(0.090)	(0.092)
**Multilevel effects**		
Year effects	Fixed	Fixed
Hospital effects	Random	Fixed
**Observations**	168 120	168 120

Data source: Yvelines district (France), 2008-2018.

Notes: * P-value < 0.05; ** P-value < 0.01; *** P-value < 0.001. Robust standard errors clustered at the hospital level in parentheses.

Table S2. Effect of hospital staffing ratios on caesareans. Multilevel logit models (coefficient estimates). All population. Assumption of 25% for part-time private doctors.

	Sample: All population					
**Dependent variable:**	**Caesarean**		**Planned caesarean**		**Unplanned caesarean**	
	(1)	(2)	(3)	(4)	(5)	(6)
**Crossed hospital staff and hospital sector variables**						
Obstetricians x Public	-0.120	-0.107	-0.654***	-0.621**	0.079	0.085
	(0.275)	(0.274)	(0.204)	(0.232)	(0.373)	(0.376)
Obstetricians x Private	0.237	0.241	0.144	-0.009	0.130	0.253
	(0.240)	(0.241)	(0.268)	(0.291)	(0.244)	(0.198)
Anesthetists x Public	0.334	0.347	0.058	0.088	0.359	0.368
	(0.246)	(0.250)	(0.195)	(0.198)	(0.259)	(0.261)
Anesthetists x Private	-0.028	-0.038	0.017	0.103	-0.076	-0.145
	(0.168)	(0.173)	(0.168)	(0.167)	(0.225)	(0.221)
Midwives x Public	-0.111	-0.116	-0.184**	-0.194**	-0.022	-0.026
	(0.166)	(0.162)	(0.063)	(0.074)	(0.207)	(0.200)
Midwives x Private	0.083	0.064	-0.186	-0.167	0.262	0.223
	(0.104)	(0.107)	(0.098)	(0.098)	(0.144)	(0.137)
**Control variables**						
Demographics	Yes	Yes	Yes	Yes	Yes	Yes
Clinical risks	Yes	Yes	Yes	Yes	Yes	Yes
Hospital type	Yes	Yes	Yes	Yes	Yes	Yes
Hospital organization	Yes	Yes	Yes	Yes	Yes	Yes
**Multilevel effects**						
Year effects	Fixed	Fixed	Fixed	Fixed	Fixed	Fixed
Hospital effects	Random	Fixed	Random	Fixed	Random	Fixed
**Observations**	168 120	168 120	168 090	168 090	150 226	150 226

Data source: Yvelines district (France), 2008-2018.

Notes: * P-value < 0.05; ** P-value < 0.01; *** P-value < 0.001. Robust standard errors clustered at the hospital level in parentheses. Control variables included for demographics: age and parity; for clinical risks: previous caesarean, diabetes, hypertension, eclampsia or preeclampsia, fetal growth restriction, placental disorder, other obstetric pathology, plurality, term at delivery, fetal presentation, induced labor, and birth weight; for hospital type: sector, equipment level, university status; and for hospital organization: obstetrician availability, day of delivery, and size.

Table S3. Effect of hospital staffing ratios on caesareans. Multilevel logit models (coefficient estimates). All population. Assumption of 75% for part-time private doctors.

Sample: All population						
**Dependent variable:**	**Caesarean**		**Planned caesarean**		**Unplanned caesarean**	
	(1)	(2)	(3)	(4)	(5)	(6)
**Crossed hospital staff and hospital sector variables**						
Obstetricians x Public	-0.118	-0.100	-0.658***	-0.616**	0.091	0.097
	(0.283)	(0.278)	(0.197)	(0.225)	(0.365)	(0.370)
Obstetricians x Private	0.066	0.044	0.065	-0.087	-0.051	0.036
	(0.134)	(0.145)	(0.221)	(0.229)	(0.174)	(0.150)
Anesthetists x Public	0.351	0.365	0.075	0.094	0.359	0.376
	(0.250)	(0.252)	(0.194)	(0.197)	(0.263)	(0.267)
Anesthetists x Private	0.022	0.032	0.008	0.101	0.011	-0.039
	(0.115)	(0.124)	(0.126)	(0.125)	(0.136)	(0.134)
Midwives x Public	-0.119	-0.123	-0.194**	-0.197*	-0.017	-0.024
	(0.171)	(0.166)	(0.063)	(0.078)	(0.212)	(0.205)
Midwives x Private	0.070	0.050	-0.181*	-0.177	0.235	0.200
	(0.126)	(0.129)	(0.097)	(0.109)	(0.203)	(0.196)
**Control variables**						
Demographics	Yes	Yes	Yes	Yes	Yes	Yes
Clinical risks	Yes	Yes	Yes	Yes	Yes	Yes
Hospital type	Yes	Yes	Yes	Yes	Yes	Yes
Hospital organization	Yes	Yes	Yes	Yes	Yes	Yes
**Multilevel effects**						
Year effects	Fixed	Fixed	Fixed	Fixed	Fixed	Fixed
Hospital effects	Random	Fixed	Random	Fixed	Random	Fixed
**Observations**	168 120	168 120	168 090	168 090	150 226	150 226

Data source: Yvelines district (France), 2008-2018.

Notes: * P-value < 0.05; ** P-value < 0.01; *** P-value < 0.001. Robust standard errors clustered at the hospital level in parentheses. Control variables included for demographics: age and parity; for clinical risks: previous caesarean, diabetes, hypertension, eclampsia or preeclampsia, fetal growth restriction, placental disorder, other obstetric pathology, plurality, term at delivery, fetal presentation, induced labor, and birth weight; for hospital type: sector, equipment level, university status; and for hospital organization: obstetrician availability, day of delivery, and size.

Table S4. Effect of hospital staffing ratios on caesareans. Multilevel logit models (coefficient estimates). High-risk population. Assumption of 25% for part-time private doctors.

Sample: High-risk population						
**Dependent variable:**	**Caesarean**		**Planned caesarean**		**Unplanned caesarean**	
	(1)	(2)	(3)	(4)	(5)	(6)
**Crossed hospital staff and hospital sector variables**						
Obstetricians x Public	-0.217	-0.181	-0.852**	-0.790*	0.077	0.088
	(0.238)	(0.249)	(0.279)	(0.322)	(0.299)	(0.318)
Obstetricians x Private	0.222	0.197	-0.189	-0.447	0.209	0.385
	(0.231)	(0.258)	(0.282)	(0.301)	(0.230)	(0.210)
Anesthetists x Public	0.226	0.269	0.035	0.098	0.228	0.260
	(0.230)	(0.231)	(0.208)	(0.210)	(0.254)	(0.255)
Anesthetists x Private	0.045	0.037	0.155	0.302	0.004	-0.138
	(0.134)	(0.147)	(0.159)	(0.130)	(0.195)	(0.193)
Midwives x Public	0.003	-0.011	-0.132*	-0.149*	0.098	0.087
	(0.149)	(0.139)	(0.066)	(0.070)	(0.195)	(0.185)
Midwives x Private	0.113	0.085	-0.096	-0.066	0.240	0.175
	(0.122)	(0.126)	(0.111)	(0.104)	(0.178)	(0.164)
**Control variables**						
Demographics	Yes	Yes	Yes	Yes	Yes	Yes
Clinical risks	Yes	Yes	Yes	Yes	Yes	Yes
Hospital type	Yes	Yes	Yes	Yes	Yes	Yes
Hospital organization	Yes	Yes	Yes	Yes	Yes	Yes
**Multilevel effects**						
Year effects	Fixed	Fixed	Fixed	Fixed	Fixed	Fixed
Hospital effects	Random	Fixed	Random	Fixed	Random	Fixed
**Observations**	82 850	82 850	82 828	82 828	68 517	68 517

Data source: Yvelines district (France), 2008-2018.

Notes: * P-value < 0.05; ** P-value < 0.01; *** P-value < 0.001. Robust standard errors clustered at the hospital level in parentheses. Control variables included for demographics: age and parity; for clinical risks: previous caesarean, diabetes, hypertension, eclampsia or preeclampsia, fetal growth restriction, placental disorder, other obstetric pathology, plurality, term at delivery, fetal presentation, induced labor, and birth weight; for hospital type: sector, equipment level,

university status; and for hospital organization: obstetrician availability, day of delivery, and size.

Table S5. Effect of hospital staffing ratios on caesareans. Multilevel logit models (coefficient estimates). High-risk population. Assumption of 75% for part-time private doctors.

Sample: High-risk population						
**Dependent variable:**	**Caesarean**		**Planned caesarean**		**Unplanned caesarean**	
	(1)	(2)	(3)	(4)	(5)	(6)
**Crossed hospital staff and hospital sector variables**						
Obstetricians x Public	-0.230	-0.188	-0.850**	-0.775*	0.067	0.082
	(0.246)	(0.252)	(0.277)	(0.326)	(0.304)	(0.322)
Obstetricians x Private	0.154	0.099	-0.143	-0.416	0.112	0.246
	(0.103)	(0.141)	(0.261)	(0.253)	(0.142)	(0.169)
Anesthetists x Public	0.249	0.291	0.027	0.077	0.242	0.283
	(0.235)	(0.232)	(0.206)	(0.212)	(0.259)	(0.259)
Anesthetists x Private	0.061	0.089	0.067	0.237	0.072	-0.020
	(0.103)	(0.119)	(0.191)	(0.147)	(0.108)	(0.105)
Midwives x Public	-0.014	-0.026	-0.128	-0.138	0.087	0.075
	(0.156)	(0.143)	(0.068)	(0.078)	(0.203)	(0.192)
Midwives x Private	0.111	0.075	-0.092	-0.096	0.219	0.163
	(0.137)	(0.139)	(0.126)	(0.134)	(0.218)	(0.207)
**Control variables**						
Demographics	Yes	Yes	Yes	Yes	Yes	Yes
Clinical risks	Yes	Yes	Yes	Yes	Yes	Yes
Hospital type	Yes	Yes	Yes	Yes	Yes	Yes
Hospital organization	Yes	Yes	Yes	Yes	Yes	Yes
**Multilevel effects**						
Year effects	Fixed	Fixed	Fixed	Fixed	Fixed	Fixed
Hospital effects	Random	Fixed	Random	Fixed	Random	Fixed
**Observations**	82 850	82 850	82 828	82 828	68 517	68 517

Data source: Yvelines district (France), 2008-2018.

Notes: * P-value < 0.05; ** P-value < 0.01; *** P-value < 0.001. Robust standard errors clustered at the hospital level in parentheses. Control variables included for demographics: age and parity; for clinical risks: previous caesarean, diabetes, hypertension, eclampsia or preeclampsia, fetal growth restriction, placental disorder, other obstetric pathology, plurality, term at delivery, fetal presentation, induced labor, and birth weight; for hospital type: sector, equipment level,

university status; and for hospital organization: obstetrician availability, day of delivery, and size.

Table S6. Effect of hospital staffing ratios on caesareans. Multilevel logit models (coefficient estimates). Low-risk population. Assumption of 25% for part-time private doctors.

Sample: Low-risk population						
**Dependent variable:**	**Caesarean**		**Planned caesarean**		**Unplanned caesarean**	
	(1)	(2)	(3)	(4)	(5)	(6)
**Crossed hospital staff and hospital sector variables**						
Obstetricians x Public	-0.107	-0.163	0.273	-0.281	-0.142	-0.202
	(0.426)	(0.421)	(0.997)	(1.307)	(0.519)	(0.534)
Obstetricians x Private	0.690	0.852*	1.425***	1.067**	0.098	0.608
	(0.433)	(0.362)	(0.324)	(0.352)	(0.557)	(0.431)
Anesthetists x Public	0.452	0.382	-0.446	-1.018**	0.586	0.551
	(0.272)	(0.260)	(0.267)	(0.329)	(0.308)	(0.294)
Anesthetists x Private	-0.208	-0.314	-0.948**	-0.751*	-0.079	-0.366
	(0.299)	(0.301)	(0.348)	(0.336)	(0.345)	(0.330)
Midwives x Public	-0.100	-0.102	0.157	0.430	-0.095	-0.161
	(0.250)	(0.228)	(0.341)	(0.365)	(0.297)	(0.245)
Midwives x Private	0.251	0.149	-0.210	-0.248	0.490	0.305
	(0.227)	(0.222)	(0.196)	(0.241)	(0.289)	(0.238)
**Control variables**						
Demographics	Yes	Yes	Yes	Yes	Yes	Yes
Clinical risks	Yes	Yes	Yes	Yes	Yes	Yes
Hospital type	Yes	Yes	Yes	Yes	Yes	Yes
Hospital organization	Yes	Yes	Yes	Yes	Yes	Yes
**Multilevel effects**						
Year effects	Fixed	Fixed	Fixed	Fixed	Fixed	Fixed
Hospital effects	Random	Fixed	Random	Fixed	Random	Fixed
**Observations**	33 370	33 370	33 369	33 369	32 271	32 271

Data source: Yvelines district (France), 2008-2018.

Notes: * P-value < 0.05; ** P-value < 0.01; *** P-value < 0.001. Robust standard errors clustered at the hospital level in parentheses. Control variables included for demographics: age and parity; for clinical risks: previous caesarean, diabetes, hypertension, eclampsia or preeclampsia, fetal growth restriction, placental disorder, other obstetric pathology, plurality, term at delivery, fetal presentation, induced labor, and birth weight; for hospital type: sector, equipment level,

university status; and for hospital organization: obstetrician availability, day of delivery, and size.

Table S7. Effect of hospital staffing ratios on caesareans. Multilevel logit models (coefficient estimates). Low-risk population. Assumption of 75% for part-time private doctors.

	**Sample: Low-risk population**					
**Dependent variable:**	**Caesarean**		**Planned caesarean**		**Unplanned caesarean**	
	(1)	(2)	(3)	(4)	(5)	(6)
**Crossed hospital staff and hospital sector variables**						
Obstetricians x Public	-0.093	-0.127	0.060	-0.342	-0.089	-0.151
	(0.435)	(0.422)	(1.018)	(1.322)	(0.495)	(0.508)
Obstetricians x Private	0.228	0.250	1.260**	0.765#	-0.237	0.050
	(0.365)	(0.376)	(0.485)	(0.490)	(0.473)	(0.477)
Anesthetists x Public	0.484*	0.431	-0.424	-0.956*	0.580	0.563
	(0.274)	(0.263)	(0.272)	(0.383)	(0.308)	(0.296)
Anesthetists x Private	-0.147	-0.173	-0.619	-0.369	-0.057	-0.218
	(0.162)	(0.165)	(0.396)	(0.405)	(0.136)	(0.162)
Midwives x Public	-0.115	-0.116	0.103	0.391	-0.067	-0.147
	(0.266)	(0.244)	(0.335)	(0.355)	(0.290)	(0.250)
Midwives x Private	0.234	0.143	-0.197	-0.232	0.472	0.297
	(0.258)	(0.261)	(0.229)	(0.253)	(0.360)	(0.340)
**Control variables**						
Demographics	Yes	Yes	Yes	Yes	Yes	Yes
Clinical risks	Yes	Yes	Yes	Yes	Yes	Yes
Hospital type	Yes	Yes	Yes	Yes	Yes	Yes
Hospital organization	Yes	Yes	Yes	Yes	Yes	Yes
**Multilevel effects**						
Year effects	Fixed	Fixed	Fixed	Fixed	Fixed	Fixed
Hospital effects	Random	Fixed	Random	Fixed	Random	Fixed
**Observations**	33 370	33 370	33 369	33 369	32 271	32 271

Data source: Yvelines district (France), 2008-2018.

Notes: * P-value < 0.05; ** P-value < 0.01; *** P-value < 0.001. # Significant at 10%. Robust standard errors clustered at the hospital level in parentheses. Control variables included for demographics: age and parity; for clinical risks: previous caesarean, diabetes, hypertension, eclampsia or preeclampsia, fetal growth restriction, placental disorder, other obstetric pathology, plurality, term at delivery, fetal presentation, induced labor, and birth weight; for

hospital type: sector, equipment level, university status; and for hospital organization: obstetrician availability, day of delivery, and size.
